# PDGFRα Enhanced Infection of Breast Cancer Cells with Human Cytomegalovirus but Infection of Fibroblasts Increased Prometastatic Inflammation Involving Lysophosphatidate Signaling

**DOI:** 10.3390/ijms22189817

**Published:** 2021-09-10

**Authors:** Zelei Yang, Xiaoyun Tang, Todd P. W. McMullen, David N. Brindley, Denise G. Hemmings

**Affiliations:** 1 Department of Biochemistry, University of Alberta, Edmonton, AB T6G 2S2, Canada; zelei@ualberta.ca (Z.Y.); xtang2@ualberta.ca (X.T.); 2 Cancer Research Institute of Northern Alberta, University of Alberta, Edmonton, AB T6G 2S2, Canada; 3 Department of Surgery, University of Alberta, Edmonton, AB T6G 2B7, Canada; todd.mcmullen@albertahealthservices.ca; 4 Cardiovascular Research Center, Medical Microbiology and Immunology, Obstetrics and Gynecology, Women and Children’s Health Research Institute, Li Ka Shing Institute of Virology, University of Alberta, Edmonton, AB T6G 2S2, Canada

**Keywords:** autotaxin, chemokines, cytokines, lipid phosphate phosphatases, lysophosphatidic acid

## Abstract

Human cytomegalovirus (HCMV) infects 40–70% of adults in developed countries. HCMV proteins and DNA are detected in tumors and metastases, suggesting an association with increased invasion. We investigated HCMV infection in human breast cancer cell lines compared to fibroblasts, a component of tumors, and the role of platelet-derived growth factor receptor-α (PDGFRα). HCMV productively infected HEL299 fibroblasts and, to a lesser extent, Hs578T breast cancer cells. Infection of another triple-negative cell line, MDA-MB-231, and also MCF-7 cells, was extremely low. These disparate infection rates correlated with expression of *PDGFRA*, which facilitates HCMV uptake. Increasing *PDGFRA* expression in T-47D breast cancer and BCPAP thyroid cancer cells markedly increased HCMV infection. Conversely, HCMV infection decreased *PDGFRA* expression, potentially attenuating signaling through this receptor. HCMV infection of fibroblasts promoted the secretion of proinflammatory factors, whereas an overall decreased secretion of inflammatory factors was observed in infected Hs578T cells. We conclude that HCMV infection in tumors will preferentially target tumor-associated fibroblasts and breast cancer cells expressing PDGFRα. HCMV infection in the tumor microenvironment, rather than cancer cells, will increase the inflammatory milieu that could enhance metastasis involving lysophosphatidate.

## 1. Introduction

Infectious agents contribute to >15% of cancers [1]. Epstein-Barr virus causes Burkitt’s lymphoma, human papillomavirus causes cervical cancer and several other viruses are associated with cancer progression. This study focuses on human cytomegalovirus (HCMV), which is a species-specific beta-herpes virus. Forty to 70% of adults in developed countries are chronically infected with HCMV, and infection increases with age [2,3]. HCMV infection is normally well tolerated except when individuals are immunocompromised [4]. Immune responses control infection; however, HCMV evades these responses to establish lifelong infections [5]. HCMV replicates in many cell types including fibroblasts and epithelial cells [6]. Viral proteins are produced during active infection in three stages: immediate early (IE), early and late, with IE proteins acting as transcriptional regulators. HCMV then enters latency where the viral genome is retained, but viral replication is very limited [7].

HCMV infection is oncomodulatory where alterations in intracellular signaling pathways promote cancer progression, but some reports also support oncogenesis [8]. HCMV DNA and/or proteins are found in glioblastoma, colorectal, prostate, ovarian and breast cancers [9]. One report claims that 95% of breast tumors, but only ~70% of breast tissues without cancer, are HCMV-positive [10]. Other studies report high detection of HCMV DNA and proteins in breast tumors [11,12], sentinel lymph nodes [11,13] and the brain [14], which indicates potential association with invasiveness and metastasis. HCMV DNA in breast tumors is also associated with poor overall survival and low relapse-free survival [15]. We showed that latent mouse CMV (mCMV) infection does not change breast tumor growth in mouse models, but the tumor characteristics are modified to promote lung metastasis without viral reactivation [16]. In contrast, mCMV infection enhances tumor growth and decreases survival in mouse models of glioblastoma with viral reactivation detected in tumor cells [17].

The susceptibility of breast cancer cells to HCMV infection is controversial. HCMV proteins are detected in breast tumors and in the cancer cells in some [10,11,13,18], but not all, studies [19,20]. The role of HCMV infection in other cells of the tumor versus breast cancer cells is unclear. HCMV enters cells through interactions of viral glycoprotein complexes with cell surface receptors such as epidermal growth factor receptor (EGFR), platelet-derived growth factor receptor-α (PDGFRα), neuropilins and integrins, depending on the cell type [6]. The present study focused on PDGFRα, which is important for HCMV infection of fibroblasts, epithelial and endothelial cells [21,22]. PDGFRα signaling is activated by HCMV glycoprotein B in glioblastomas, promoting survival and motility of glioma cells [23]. This receptor is also associated with increased metastasis and mortality independently of infection in thyroid and breast cancer [24,25,26]. However, little is known about the importance of PDGFRα expression on HCMV infection in breast cancer cells.

Chronic HCMV infection intersects with many hallmarks of cancer, including tumor-promoting inflammation [9,27]. Recognition of HCMV by TLR2 and CD14 triggers inflammatory cytokine production through NF-κB [28], and this is enhanced by HCMV IE transactivation [29]. Pro-inflammatory mediators such as interleukin (IL)-6 and prostaglandin-endoperoxide synthase 2 (PTGS2, cyclooxygenase-2) promote cancer progression [30], and can be produced by HCMV infection [31]. Inflammatory mediators from breast tumors also stimulate autotaxin (ATX) secretion from adjacent adipose tissue, which produces lysophosphatidate (LPA) [32]. LPA receptor signaling promotes further inflammation that leads to cancer progression, metastasis and immune evasion [32]. HCMV infection increases ATX expression in human trabecular meshwork cells [33], but the impact in breast tumors is not known. HCMV-encoded IL-10 (cmvIL-10) also stimulates proliferation, migration and invasion of breast cancer cells in vitro [34,35]. However, whether inflammation or cmvIL-10 is induced by HCMV-infected breast cancer cells or by other infected cells in the tumor is unknown.

The present study investigated HCMV infectivity and production of inflammatory mediators in four human breast cancer cell lines compared to human fibroblasts. Infectivity and productive infection of breast cancer cell lines with a low passage clinical HCMV isolate depended on the level of PDGFRα expression. HCMV infection of HEL299 fibroblasts resulted in a pro-inflammatory environment, whereas infection of PDGFRα-positive Hs578T cells did not. We conclude that HCMV infection in breast tumors will occur mainly in tumor-associated fibroblasts and in breast cancer cells expressing PDGFRα. HCMV-induced inflammation in the tumor microenvironment involving LPA signaling could contribute to the observed increase in metastasis and mortality.

## 2. Results

### 2.1. IE and CmvIL-10 Expression in Human Fibroblasts and Breast Cancer Cells

**Figure 1:**
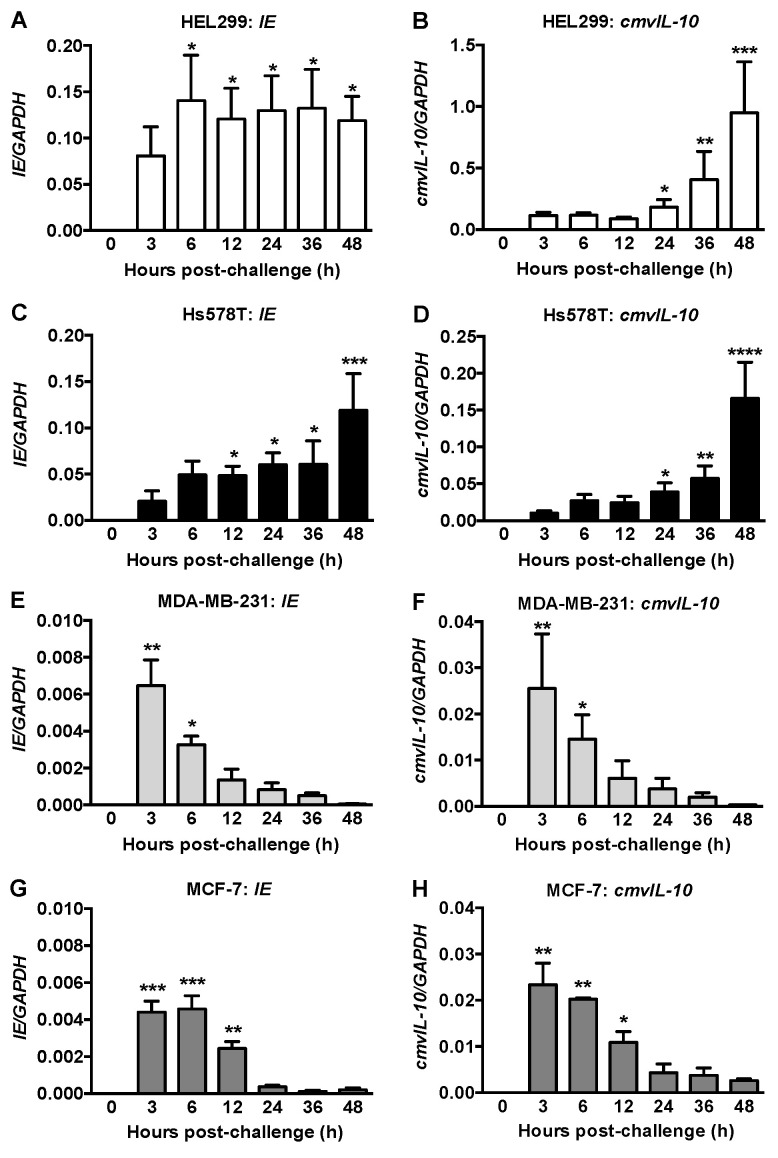
mRNA expression of viral genes in human fibroblasts and breast cancer cells. HEL299 fibroblasts (**A**,**B**) and Hs578T (**C**,**D**), MDA-MD-231 (**E**,**F**) and MCF7 (**G**,**H**) breast cancer cells were exposed to 1 (**A**,**B**) or 2 MOI (**C**–**H**) of HCMV for the times indicated. mRNA of immediate early (IE; **A**,**C**,**E**,**G**) and cmvIL-10 (**B**,**D**,**F**,**H**) viral genes were measured and normalized to GAPDH. No expression of viral mRNA was detected at 0 h. Results are mean ± SEM from 3–5 independent experiments and *p*-values were calculated by the Kruskal-Wallis test with a Dunn’s multiple comparisons test. *, *p* < 0.05; **, *p* < 0.01; ***, *p* < 0.001; ****, *p* < 0.0001.

Fibroblasts constitute part of breast tumors and are highly susceptible to CMV infection [6]. We first cultured Hs578Bst cells, a fibroblast-like cell line that was derived from the same patient as Hs578T breast cancer cells. However, we were unable to obtain sufficient numbers of healthy cells even after purchasing new cells and employing several culture conditions including the recommended Hybri-Care Medium (ATCC, Manassas, VA, USA) supplemented with 30 ng/mL mouse EGF and 10% FBS. HEL299 fibroblasts were used instead and were exposed to 1 Multiplicity of Infection (MOI) of HCMV. Expression of *IE* and *cmvIL-10* mRNAs were detected after 3 h with significant increased levels of *IE* from 6–48 h (Figure 1A) and *cmvIL-10* from 24–48 h (Figure 1B) compared to 0 h.

Hs578T, MDA-MB-231 and MCF-7 breast cancer cells were exposed to 2 MOI of HCMV. Expression of *IE* and *cmvIL-10* mRNAs in Hs578T cells was significantly detected at 12 h or 24 h, respectively, and increased at 48 h compared to 0 h (Figure 1C,D). MDA-MB-231 and MCF-7 cells showed ~five-fold less relative mRNA expression for *IE* and *cmvIL-10* under the same conditions. The highest expressions were observed between 3–6 h in MDA-MB-231 and 3–12 h in MCF-7 cells and these decreased substantially thereafter (Figure 1E,H).

**Figure 2:**
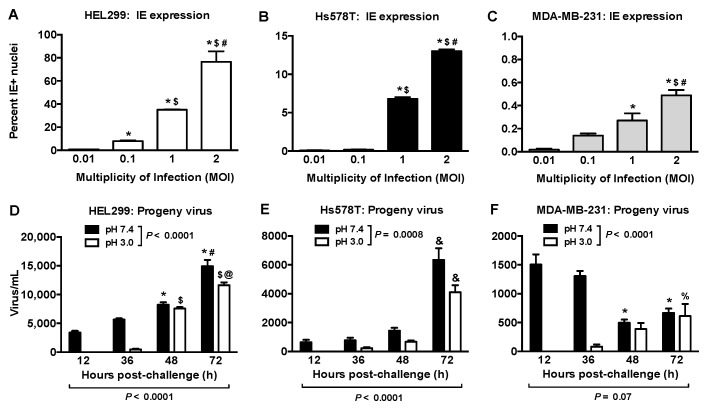
Immediate early (IE) antigen expression and production of progeny virus differed in HCMV-challenged fibroblasts and breast cancer cells. (**A**–**C**), HEL299 fibroblasts (**A**), Hs578T (**B**) and MDA-MB-231 (**C**) breast cancer cells were exposed to different HCMV MOIs and cells positive for IE antigen were quantified 36 h later. Results are mean ± SEM from three independent experiments. *P-*values were calculated by one-way ANOVA with a Tukey’s post-hoc test. *, *p* < 0.01 compared to 0.01 MOI; $, *p* < 0.01 compared to 0.1 MOI; #, *p* < 0.05 compared to 0.01 MOI; $, *p* < 0.01 compared to 0.1 MOI; #, *p* < 0.05 compared to 1 MOI. (**D**–**F**), Infectious virus was quantified from cell lysates after exposure to 0.1 MOI in HEL299 fibroblasts (**D**) or 2 MOI in breast cancer cells (**E**,**F**). Cells were washed 2 h after viral exposure with HEPES-buffered saline at pH 7.4 or pH 3.0 followed by further culture in normal media until collection of cell lysates at the times indicated. Lysates were added to fresh uninfected HEL299 fibroblasts for 24 h and cells were stained for HCMV IE antigen denoting infectious virus in the lysates, with results expressed as virus/mL. Results are means ± SEM from three independent experiments with *p*-values calculated by two-way ANOVA and Tukey’s post hoc test. *, *p* < 0.05 compared to 12 and 36 h pH 7.4 wash; $, *p* < 0.0001 compared to 12 and 36 h pH 3.0 wash; #, *p* < 0.01 compared to 48 h pH 7.4 wash; @, *p* < 0.05 compared to 48 h pH 3.0 wash; &, *p* < 0.0001 compared to 12, 36, and 48 h washes; %, *p* < 0.05 compared to 12 h pH 3.0 wash.

IE-positive nuclei at 36 h post viral exposure increased with MOI in all cells tested (Figure 2A–C and Figure S1). At 2 MOI, ~80% of the HEL299 fibroblasts expressed IE antigen (Figure 2A) compared to ~13% for Hs578T cells (Figure 2B). MDA-MB-231 and MCF-7 cells showed IE-positive nuclei <1%, (Figure 2C, Figures S1 and S2A). Cells exposed to the filtered control did not express IE antigen (Supplementary Figure S1).

### 2.2. Productive HCMV Infection in Hs578T, MDA-MB-231 and MCF-7 Breast Cancer Cells

We compared the production of progeny virus in breast cancer cells to that in HEL299 fibroblasts at 2 or 0.1 MOI, respectively. HEL299 fibroblasts washed with HEPES-buffered saline (HBS) at pH 7.4 had cell lysates that contained ~5000 infectious virus/mL at 12 and 36 h (black bars; Figure 2D). This occurred before expected viral production, suggesting that input virus remained on the cell surface. Washing at pH 3.0, which removes surface-bound but not internalized virus [36], eliminated infectious virus levels at 12 and 36 h, whereas progeny virus were produced at 48 and 72 h (white bars; Figure 2D).

HEL 299 fibroblasts infected with 0.1 MOI of HCMV produced ~7500 and ~14,000 virus/mL at 48 h and 72 h (Figure 2D). Hs578T cells produced ~4000–6000 virus/mL after 72 h (Figure 2E). MDA-MB-231 and MCF-7 cells produced very few progeny virus (Figure 2F and Figure S2B).

### 2.3. HCMV IE Expression Level Is Correlated with PDGFRA Expression

**Figure 3:**
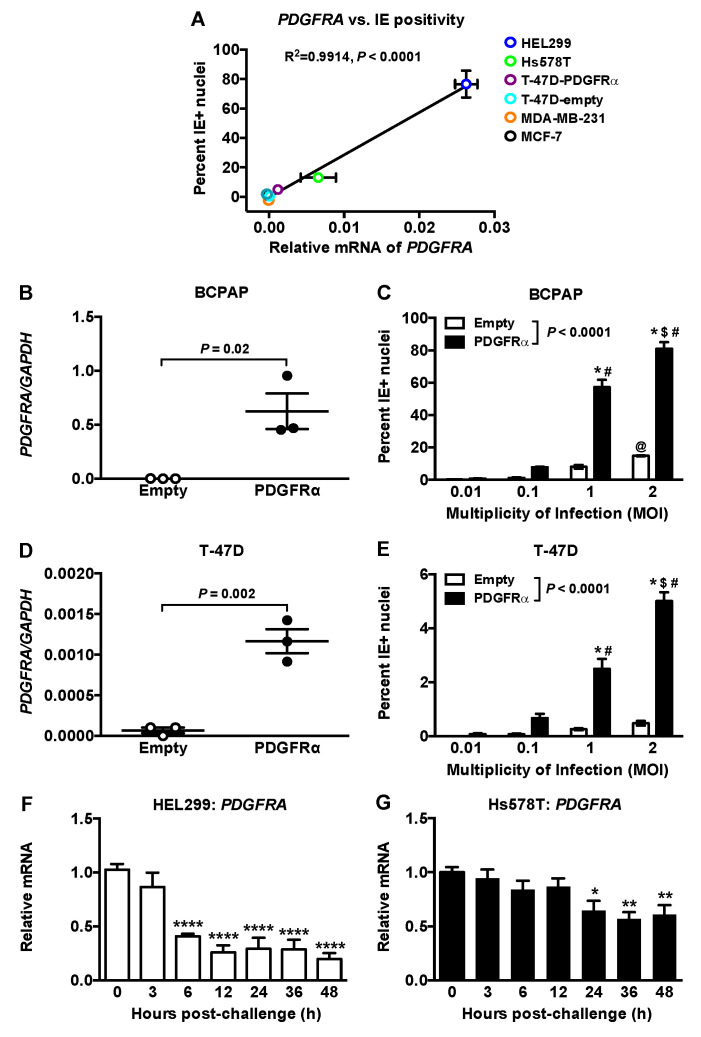
HCMV infection correlated with PDGFRA expression. (**A**) Regression analysis of PDGFRA mRNA versus percent IE antigen positive nuclei following exposure to 2 MOI of HCMV in HEL299 fibroblasts and breast cancer cells. (**B**,**D**) PDGFRA mRNA expression in BCPAP thyroid cancer cells and T-47D breast cancer cells transduced with an empty vector or PDGFRA under an inducible promoter. Results are mean ± SEM from three independent experiments and P-values were calculated by unpaired Student’s *t*-test. (**C**,**E**) Cell lines were exposed to 0.01, 0.1, 1 or 2 MOI of HCMV and stained for IE at 36 h later. Results are means ± SEM from three independent experiments. *p*-values were calculated by two-way ANOVA and Tukey’s post hoc test. *, *p* < 0.0001 compared to 0.01 and 0.1 MOI challenged PDGFRα expressing cells; $, *p* < 0.0001 compared to 1 MOI challenged PDGFRα expressing cells; #, *p* < 0.0001 compared to empty vector cells challenged with the same MOI; @, *p* < 0.05 compared to 0.01 and 0.1 MOI challenged empty vector cells. (**F**) and (**G**) HEL299 fibroblasts (**F**) and Hs578T breast cancer cells (**G**) were exposed to 1 or 2 MOI of HCMV, respectively. PDGFRA mRNA was quantified in lysates and expressed relative to PDGFRA mRNA in lysates from cells treated with a filtered control at each timepoint, and then normalized to 0 h. Results are means ± SEM from five independent experiments and P-values were calculated by two-way ANOVA with Tukey’s post-hoc test. *, *p* < 0.05; **, *p* < 0.01; ****, *p* < 0.0001.

The expression of *PDGFRA* mRNA and percent IE antigen positivity following exposure to 2 MOI of HCMV were highly correlated among cell lines (Figure 3A). *PDGFRA* mRNA was undetectable in MDA-MB-231 and MCF-7 cells as expected from the Cancer Cell Line Encyclopedia [37]. We studied the effects of over-expressing PDGFRα protein in BCPAP thyroid cancer cells and T-47D breast cancer cells [24]. Increased *PDGFRA* mRNA expression was paralleled by increased IE antigen positivity (Figure 3B–E).

We determined if HCMV infection altered *PDGFRA* expression because the PDGFRα is associated with increased metastasis [23,24,25,26]. *PDGFRA* mRNA was decreased by ~50% in HEL299 fibroblasts and Hs578T cells at 6 h and 24 h post viral challenge, respectively (Figure 3F,G). *EGFR* mRNA did not change following HCMV exposure (Supplementary Figure S3).

### 2.4. HCMV Infection of Fibroblasts and Breast Cancer Cells Differentially Modified the Expressions of Inflammatory Mediators and ATX/LPA Signaling

**Figure 4:**
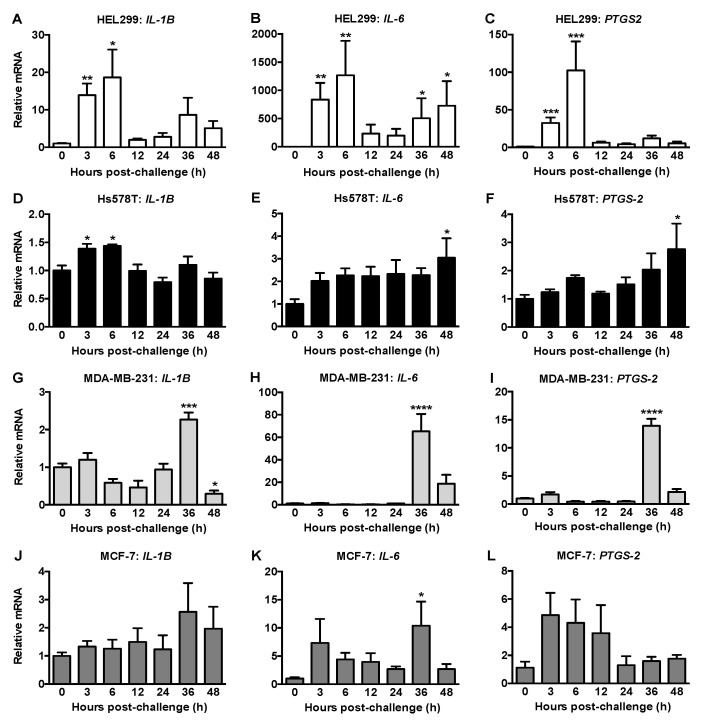
Proinflammatory mRNA expression was differentially increased in HEL299 fibroblasts and breast cancer cells exposed to HCMV. HEL299 fibroblasts (**A**–**C**) were exposed to HCMV at 1 MOI and Hs578T (**D**–**F**), MDA-MB-231 (**G**–**I**) and MCF-7 (**J**–**L**) breast cancer cells were exposed to HCMV at 2 MOI. Lysates were collected and mRNA expression was measured. Results are expressed relative to lysates from cells treated with a filtered control at each timepoint and then normalized to 0 h. Results are means ± SEM from 3–5 independent experiments and *p*-values were calculated by the nonparametric Kruskal-Wallis test with a Dunn’s multiple comparisons test (**A**–**C**) or by one-way ANOVA if the analyses passed the Brown Forsythe test (**D**–**L**). *, *p* < 0.05; **, *p* < 0.01; ***, *p* < 0.001; ****, *p* < 0.0001.

*IL-1β*, *IL-6* and *PTGS2* mRNA levels increased dramatically in the HEL299 fibroblasts predominantly at 3 and 6 h post-HCMV exposure (Figure 4A–C). For Hs578T cells, mRNA for *IL-1β* was increased at 3 and 6 h, whereas mRNAs for *IL-6* and *PTGS2* were only increased at 48 h (Figure 4D–F). For MDA-MB-231 cells, *IL-1β*, *IL-6* and *PTGS2* mRNAs were increased at 36 h post viral challenge followed by decreases at 48 h (Figure 4G–I). Although mRNA for *IL-6* was increased at 36 h in HCMV-exposed MCF-7 cells, mRNA for *IL-1β* and *PTGS2* mRNA did not change (Figure 4J–L).

We tested whether the modest mRNA changes in MDA-MB-231 cells required transcription of HCMV viral genes by treatment with UV-inactivated HCMV, which can bind to the cells but cannot generate viral transcripts. Treatment with UV-inactivated virus prevented the increase of *IL-1β* and the mRNA expression of *IE* and *cmvIL-10* at all MOIs (Supplementary Figure S4).

**Figure 5:**
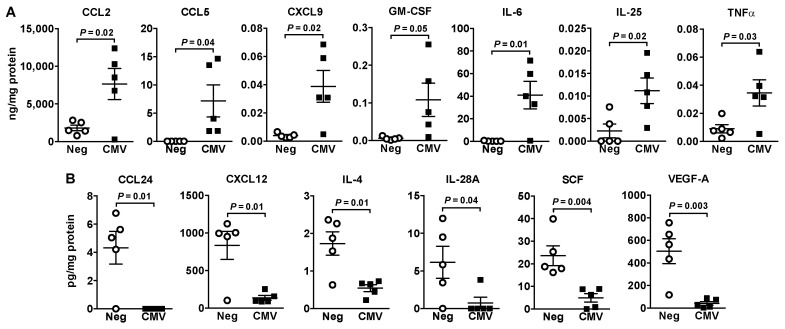
Secretion of pro and anti-inflammatory cytokines/chemokines from infected and uninfected HEL299 fibroblasts. Cells were exposed to 1 MOI HCMV or filtered control (Neg) for 48 h. The concentrations of 71 cytokines/chemokines/growth factors were analyzed in the culture media and those significantly increased (**A**) or decreased (**B**) are illustrated. Results are means ± SEM from five independent experiments. *p*-values were calculated by unpaired Student’s *t*-test.

We also analyzed the secretions of 71 cytokines, chemokines and growth factors at 48 h post-HCMV exposure. For HEL299 fibroblasts, seven proinflammatory cytokines/chemokines were increased (Figure 5A) while six anti-inflammatory or anti-tumor factors were decreased (Figure 5B). G-CSF (*p* = 0.06), IL-8 (*p* = 0.08) and IFNγ (*p* = 0.06) also tended to increase (Supplementary Table S2). Apart from 17 factors that were undetected, no changes were observed for the remaining 41 (Supplementary Table S2).

**Figure 6:**
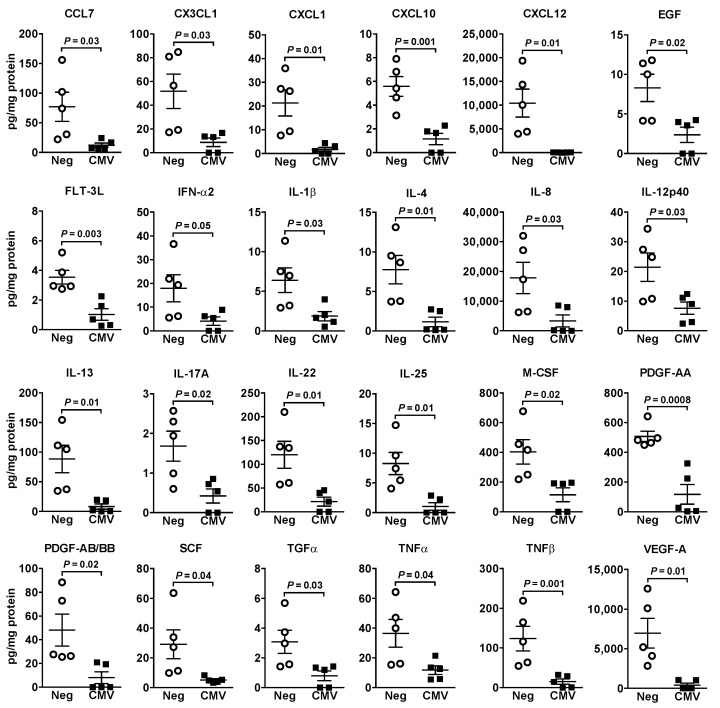
Pro-inflammatory cytokine secretion was decreased after exposing Hs578T breast cancer cells to HCMV. Cells were exposed to 2 MOI HCMV or filtered control (Neg) for 48 h. Concentrations of 71 cytokines/chemokines/growth factors were analyzed in the culture media and those with significant changes are illustrated. Results are means ± SEM from five independent experiments and *p*-values were calculated by unpaired Student’s *t*-test. Abbreviations: EGF, epidermal growth factor; FLT-3L, FMS-like tyrosine kinase 3 ligand; IFN, interferon; TGF, transforming growth factor.

HCMV-induced changes in cytokine/chemokine/growth factor secretions from Hs578T breast cancer cells differed from those in HEL299 fibroblasts. Twenty-four factors decreased after exposure to HCMV (Figure 6) with trends towards a decrease in CCL26 (*p* = 0.08) and IL-15 (*p* = 0.07) (Supplementary Table S3). Apart from 21 factors that were undetected, no changes were observed for the remaining 24 (Supplementary Table S3). In MDA-MB-231 cells, only IL-17F was increased (~6.5-fold; *p* = 0.02) and there was a trend towards a decrease in CXCL5 (*p* = 0.08) (Supplementary Table S4). No changes occurred in 34 cytokines/chemokines/growth factors and the rest were undetectable.

**Figure 7:**
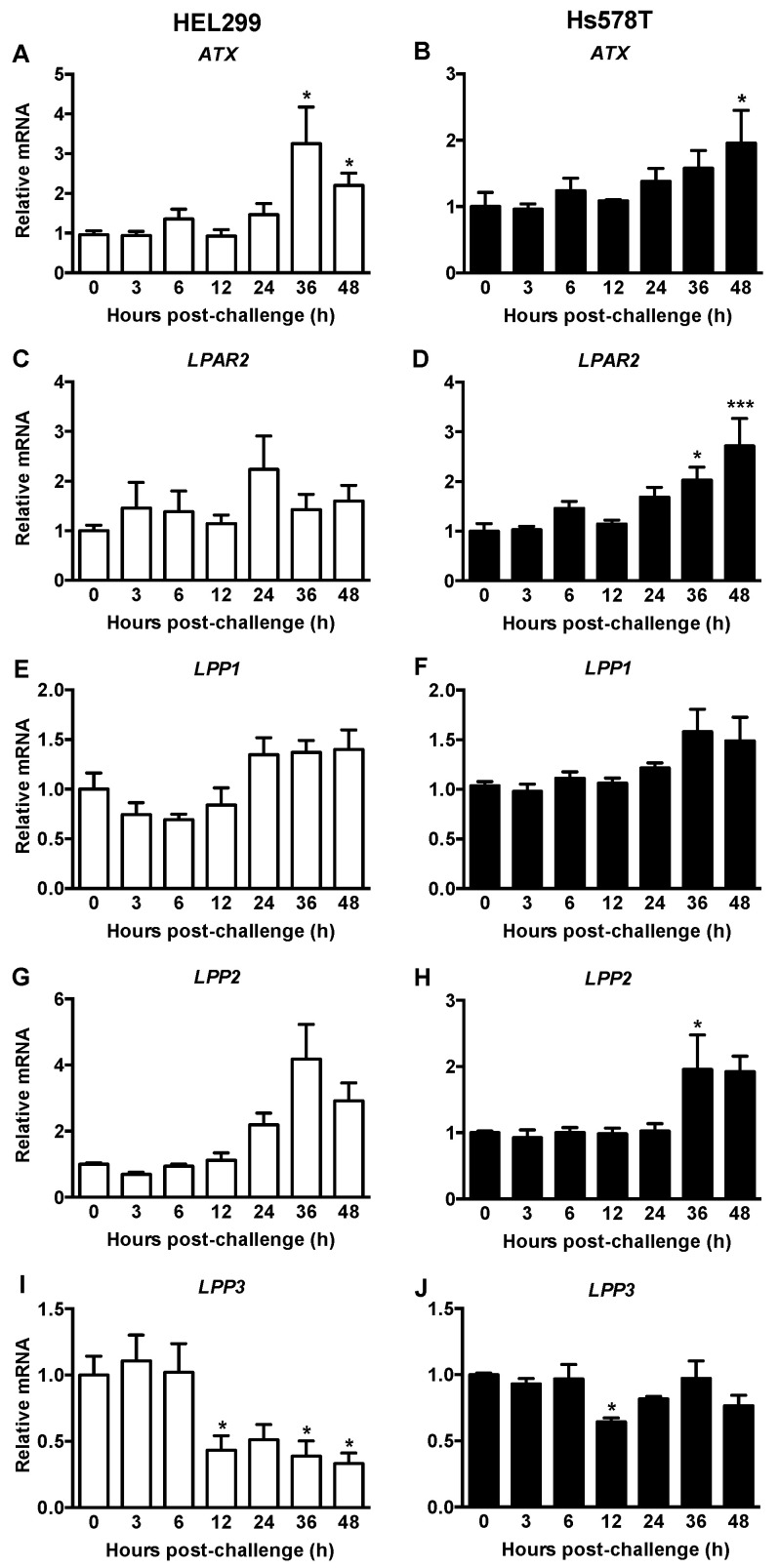
Exposure of HEL299 fibroblasts and Hs578T breast cancer cells to HCMV changed mRNA expression of genes involved in ATX/LPA signaling. HEL299 fibroblasts (**A**,**C**,**E**,**G**,**I**) and Hs578T breast cancer cells (**B**,**D**,**F**,**H**,**J**) were exposed to 1 or 2 MOI of HCMV, respectively. Lysates were collected and mRNAs were measured. Results are expressed relative to cells treated with a filtered control at each timepoint, and then normalized to 0 h. Results are means ± SEM from five independent experiments. *p*-values were calculated by the nonparametric Kruskal-Wallis test with a Dunn’s multiple comparisons test (**A**,**G**) or by one-way ANOVA if the analyses passed the Brown Forsythe test (**B**–**F**,**H**–**J**). *, *p* < 0.05; ***, *p* < 0.001.

ATX/LPA signaling drives inflammation that protects breast tumor growth and facilitates metastasis [32]. We, therefore, determined if HCMV infection alters ATX/LPA signaling. mRNA expression for *ATX* was increased by ~three-fold in HEL299 fibroblasts and ~two-fold in Hs578T cells at 36 and 48 h post-viral exposure, respectively (Figure 7A,B). Despite increased *ATX* mRNA expression, ATX activity in culture media was not changed or decreased from HEL299 fibroblasts or Hs578T cells, respectively, at 48 h post viral exposure. ATX activity increased back to the control levels by 72 h in Hs578T cells (Supplementary Figure S5). mRNA and ATX activity were barely detectable in MDA-MB-231 and MCF-7 cells, as expected [32].

Expression of mRNA for the *LPA_2_* receptor (*LPAR2*) did not change in HCMV-exposed HEL299 fibroblasts, but it gradually increased in the Hs578T cells reaching significance at 36 and 48 h (Figure 7C,D). *LPAR1* and *LPAR3* were below detection limits and no change was observed in mRNA expression for lipid phosphate phosphatase 1 (*LPP1*) in either cell type (Figure 7E,F). There was a trend towards an increase in *LPP2* mRNA in HEL299 fibroblasts and this was significant in Hs578T cells at 36 h post viral exposure (Figure 7G,H). *LPP3* mRNA expression decreased by ~50% in HEL299 fibroblasts from 12–48 h post-viral exposure, whereas it only decreased at 12 h in Hs578T cells (Figure 7I,J).

## 3. Discussion

HCMV is detected in breast tumors, but direct effects of HCMV infection on breast cancer cells still requires investigation. It is important to understand the consequences of HCMV infection in breast cancer cells compared to fibroblasts in the tumor, since each play different roles in cancer progression. We found that fibroblasts were highly susceptible to HCMV infection compared to four breast cancer cell lines. Differential infection levels were highly correlated to PDGFRα expression. Infection of fibroblasts preferentially enhanced the production of inflammatory mediators including signaling by LPA compared to breast cancer cells.

Hs578T cells were productively infected by a low passage clinical isolate of HCMV but considerably less than in HEL299 fibroblasts. Infectivity and production of progeny virus were extremely limited in MDA-MB-231 cells, which are triple-negative like Hs578T cells, and in infected MCF-7 cells after 72 h. However, higher infectivity was reported in these cell lines exposed to HCMV for >4 days [18,31,38] and/or at high MOIs of 5–10 [31,38]. Similar to the current study, infection in these cell lines appears to be very limited [18,38]. Differences in infectivity could also be related to the viral strain and the cell type for viral passage. While the current study used a clinical isolate passaged in fibroblasts, other studies used TB40/E-GFP passaged in fibroblasts [18], VR1814 passaged in endothelial cells [31] or TB40/E passaged in epithelial cells [38]. Extensive passage in fibroblasts can decrease cell entry because of mutations in the virion envelope glycoprotein complex [39]. To mitigate this, we used a clinical isolate at a low passage number.

HCMV uptake depends on its interaction with various receptors [6]. We focused on PDGFRα because its activation creates a metastatic phenotype including an increased migratory potential, which is linked to metastases in papillary thyroid cancer [24,25] and breast cancer [26]. In addition, over-expression of PDGFRα increases HCMV entry into epithelial and endothelial cells that are low or non-permissive for HCMV [6], although this is an abnormal pathway that does not involve direct interaction between HCMV and PDGFRα [40]. Our work shows a strong correlation between *PDGFRA* expression and IE antigen detection after viral exposure in HEL299 fibroblasts and breast cancer cell lines. A causal role in HCMV infection was established because overexpressing *PDGFRA* in T-47D breast cancer and BCPAP thyroid cancer cells substantially increased infectivity.

Significantly, exposure of HEL299 fibroblasts and Hs578T breast cancer cells to HCMV decreased mRNA expression for *PDGFRA*, whereas there was no effect on *EGFR* mRNA. This is compatible with the downregulation of PDGFRα by HCMV infection of coronary artery smooth muscle cells and fibroblasts [41]. However, this downregulation could mean reduced signaling through PDGFRα. This is surprising because activation of PDGFRα by glycoprotein B in glioblastomas [23] increases cancer progression and metastases [23,24,25,26]. In addition, HCMV infection of Hs578T cells decreased secretion of PDGF-AA/AB/BB, which are PDGFRα ligands, but these were not changed in infected HEL299 cells. In contrast, HCMV infection of vascular cells increases expression of PDGF-AA [42].

Oncomodulation by HCMV infection includes regulating tumor-promoting inflammation [8]. IE proteins transactivate NF-κB followed by increased cytokines/chemokines production [29]. Increased mRNA expression of three inflammatory genes after exposure of HEL299 fibroblasts to HCMV is compatible with other studies [5,31]. Increased secretion of IL-6, TNFα, CCL2, CCL5, CXCL9 and GM-CSF, which are pro-inflammatory factors combined with decreased secretion of anti-inflammatory factors including IL-4, IL-28A, CCL24, CXCL12 and SCF from infected HEL299 fibroblasts, is expected to be tumor-promoting and enhance metastasis [30,43,44]. Both IL-28A and SCF produce antitumor effects, and higher levels of CCL24 improve prognosis in breast cancer [30,44,45]. This emphasizes the critical role that HCMV infection of tumor-associated fibroblasts could play in supporting tumor growth and metastasis and its role in perpetuating a feed forward loop through activation of ATX/LPA by inflammation [32].

HCMV infection of Hs578T breast cancer cells produced a different pattern with minimally increased mRNA expression of three proinflammatory mediators, and either decreased or no change in the secreted factors. This included decreased anti-inflammatory factors such as IL-4, IL-13, M-CSF and CXCL12 [30] and antitumor factors SCF, FLT-3L and IL-25 [43,46,47] that could contribute to cancer progression and metastasis. However, growth factors including EGF, PDGF-AA, PDGF-AB/BB, and VEGF-A and several pro-inflammatory proteins, including IL-1β, IL-8, TNFα, CXCL1, CXCL10 and CX3CL1 [30,44,48] were also decreased in HCMV-exposed Hs578T cells. Overall, these findings indicate that the proinflammatory milieu, which promotes tumor growth and metastasis, should originate mainly from tumor-associated fibroblasts rather than from breast cancer cells. However, infected cancer cells might contribute by reducing production of antitumor cytokines/chemokines.

Although the expressions of IE antigen and *cmvIL-10* were very limited and short-lived in MDA-MB-231 and MCF-7 breast cancer cells exposed to HCMV, increased mRNA expression of proinflammatory mediators was observed, and this required viral entry and transcription. This was probably caused by input virus since IE antigen and *cmvIL-10* are immediately transcribed when HCMV enters a susceptible cell [49] and infection of these cells is likely abortive, as we and others showed [38]. This is compatible with the increased *PTGS2* expression observed in these cells at higher MOIs of HCMV [31]. Thus, HCMV can alter cytokine expression in cancer cells even in the absence of a robust infection.

ATX derived from adipocytes or tumor-associated fibroblasts drives tumor-promoting inflammation in breast cancer because most breast cancer cells, apart from Hs578T cells, express little ATX [32]. ATX produces LPA, which activates LPA receptors on cancer cells to increase the expression of PTGS2 and inflammatory cytokines/chemokines. *ATX* mRNA was elevated in HEL299 fibroblasts and Hs578T cells at 36 and 48 h after exposure to HCMV. These late effects probably resulted secondary to the production of inflammatory mediators [32] as illustrated by the earlier increase in mRNA for *IL-6*, *PTGS2* and *IL-1β* in fibroblasts and *IL-1β* in Hs578T cells. Significantly, ATX activity in the media collected from Hs578T cells was decreased between 0–48 h after initial infection with HCMV, but recovered to control levels after 72 h. These results indicate a late recovery of LPA production could increase signaling, especially through LPAR2 for which expression increased in Hs578T cells at 48 h post viral exposure.

The LPP family dephosphorylate extracellular LPA and so they are important regulators of LPA binding to receptors and downstream signaling [50]. LPP1 and LPP3 expressions typically decrease in cancer cells and tumors, and this increases cell division and migration [50]. *LPP3* mRNA expression was decreased following HCMV exposure. By contrast, LPP2 expression increases in cancer cells with a reduced S-phase of the cell cycle and increased anchorage-independent growth [50]. *LPP2* mRNA expression was increased in Hs578T cells at 36 h following HCMV exposure. An HCMV-induced increase in *LPP2* expression relative to *LPP3* indicates a more transformed phenotype.

The present studies investigated HCMV infectivity of a human fibroblast and four breast cancer cell lines and its impact on the production of cytokines/chemokines/growth factors. Hs578T cells underwent a productive viral infection when exposed to HCMV, as did HEL299 fibroblasts that depended on the expression of *PDGFRA*. MDA-MB-231 and MCF-7 cells did not express *PDGFRA* and showed low infectivity. The increased production and secretion of proinflammatory mediators by infected HEL299 fibroblasts along with decreased secretion of anti-inflammatory cytokines/chemokines implicates HCMV infection of tumor-associated fibroblasts in creating prometastatic conditions in the tumor microenvironment. This includes changes in LPA signaling components that üüa more inflammatory and metastatic phenotype. In contrast, infection of most breast cancer cells by HCMV, especially those with low PDGFRα expression, does not contribute to the pro-inflammatory environment through cytokine/chemokine/growth factor release. The present work demonstrates that HCMV preferentially infects fibroblasts, and this can increase the inflammatory milieu of the tumor including LPA signaling that can trigger tumor progression and metastasis.

## 4. Materials and Methods

### 4.1. Cell Culture and Reagents

The following cell lines were used: human embryonic lung 299 fibroblasts (HEL299, ATCC CCL-137, Manassa, VA, USA), Hs578Bst breast stromal cells (ATCC HTB-125), BCPAP human thyroid cancer cells (DSMZ ACC-273, German Collection of Microorganisms and Cell Cultures, Braunschweig-Süd, Germany) and human breast cancer cells Hs578T (ATCC HTB-126), MDA-MB-231 (ATCC HTB-26), MCF-7 (ATCC HTB-22) and T-47D (ATCC HTB-133). Transduction to express PDGFRα under a doxycycline-inducible promoter has been described [24]. Cells were used within 15 passages and tested negative for mycoplasma (MycoAlert Mycoplasma Detection kit, Lonza, Allendale, NJ, USA). DMEM and FBS were from Life Technologies (Grand Island, NY, USA). Primers were from Integrated DNA Technologies Inc. (Coralville, IA, USA). Other reagents were from Sigma-Aldrich (Oakville, ON, Canada).

### 4.2. Infection with HCMV

A clinical isolate of HCMV, Kp7, was obtained from J.K. Preiksaitis, University of Alberta, Edmonton, propagated in HEL299 fibroblasts [36] and used at <8 passages. A filtered control was generated by passage through a 0.05 μm membrane filter (Millipore Sigma, Tullagreen, Carrigtwohill Co. Cork, Ireland). UV-inactivated HCMV was generated by irradiation for 30 min with a 230-volt UV lamp at 20 cm.

Cells were cultured with DMEM containing 10% FBS until ~80% confluent and washed twice with HBS. Serum-free DMEM containing control solutions or active HCMV were then added as calculated by [Multiplicity of Infection (MOI) × Cells per well]/Titre of Virus. Nonattached viral particles were removed after 2 h by washing three times with serum-free DMEM. Cells were further incubated with DMEM containing 0.1% fatty acid-free BSA (for mRNA and secreted factor analysis) or 1% FBS (for productive infection experiments).

### 4.3. Quantitative Real-Time PCR (qRT-PCR)

RNA was isolated using an ultRNA Column Purification kit (Applied Biological Materials, Richmond, BC, Canada). cDNA was generated using the 5X All-In-One Reverse Transcription MasterMix (Applied Biological Materials, Richmond, BC, Canada). qRT-PCR was performed using EvaGreen qPCR master mix (Applied Biological Materials) in the Applied Biosystems 7500 real-time RT-PCR system (Life Technologies, Grand Island, NY, USA). The relative abundance of target genes was normalized to glyceraldehyde 3-phosphate dehydrogenase (*GAPDH*), with HCMV-exposed cells normalized against cells exposed to the filtered control at each time point, when applicable. Primers are listed in Supplementary Table S1.

### 4.4. Immunohistochemistry

Cells were washed three times with HBS, fixed with methanol for 10 min at −20 °C and washed further prior to blocking with 10% normal goat serum. Visualization of binding by anticytomegalovirus primary antibody, clone 8B1.1 diluted 1:500 (Millipore, MAB810) was performed using the Histostain^®^-Plus Bulk Kit followed by the AEC Single Solution kit (Invitrogen, Carlsbad, CA, USA). Colored images were taken with EVOS XL Core Imaging System (Invitrogen, Carlsbad, CA, USA) at 20× magnification and phase contrast images were taken with AMG EVOS FL Digital Inverted Microscope (Fisher Scientific, Rockford, IL, USA) at 10X magnification. IE-positive nuclei were averaged from five random fields per well and the average from three wells was determined in each experiment.

### 4.5. Determination of Productive HCMV Infection

HEL299 fibroblasts and human breast cancer cells were exposed to 0.1 or 2 MOI of HCMV, respectively. Some cultures were washed three times after 2 h for 20 s each with HBS, pH 7.4, to remove nonadherent virus. Other cultures were washed similarly with HBS, pH 3.0, to release viral particles attached to the cell surface. Neither wash affected internalized virus or cell integrity. After five washes with HBS, pH 7.4, cells were cultured in DMEM containing 1% FBS for 72 h. Combined cells and culture media were frozen and thawed three times, with vigorous pipetting to facilitate cell lysis and release of viral progeny. Serial dilutions of lysates were added to uninfected HEL299 fibroblasts and cells were stained for IE after 24 h. IE-positive nuclei were expressed as virus/mL based on lysate volume, representing infectious HCMV viral particles [36].

### 4.6. Analysis of Cytokines/Chemokines/Growth Factors

Culture media were collected at 48 h post-HCMV exposure to coincide with release of progeny virus. Media were centrifuged and filtered (0.05 µm pore size) to remove viral particles prior to blinded analysis using the Human Cytokine Array/Chemokine Array 71-plex ELISA Discovery Luminex assay by Eve Technologies Corp (Calgary, AB, Canada). Concentrations were normalized to volume and total cell protein concentration in each well (BCA protein assay, Thermo Fisher Scientific, Rockford, IL, USA).

### 4.7. Measurement of ATX Activity

ATX activity in culture media (30 μL) was measured by choline release from lysophosphatidylcholine [51].

### 4.8. Statistical Analyses

Statistical analyses were conducted using GraphPad Prism 6 (La Jolla, CA, USA) as described in each Figure legend. *p* < 0.05 were considered significant.

## Data Availability

Data concerning expression of PDGFRα available at Cancer Cell Line Encyclopedia. Available online: https://portals.broadinstitute.org/ccle/page?gene=PDGFRA accessed on 6 September 2021.

## Supplementary Materials

The following are available online at https://www.mdpi.com/article/10.3390/ijms22189817/s1.
