# Supplementary material for: PDGFRα Enhanced Infection of Breast Cancer Cells with Human Cytomegalovirus but Infection of Fibroblasts Increased Prometastatic Inflammation Involving Lysophosphatidate Signaling
Source: Int J Mol Sci. 2021 Sep 10;22(18):9817. doi: 10.3390/ijms22189817 (PMC8471290; doi:10.3390/ijms22189817)
Supplement: Supplementary file 1 [file ijms-22-09817-s001.zip › ijms-1363940-supplementary.pdf]

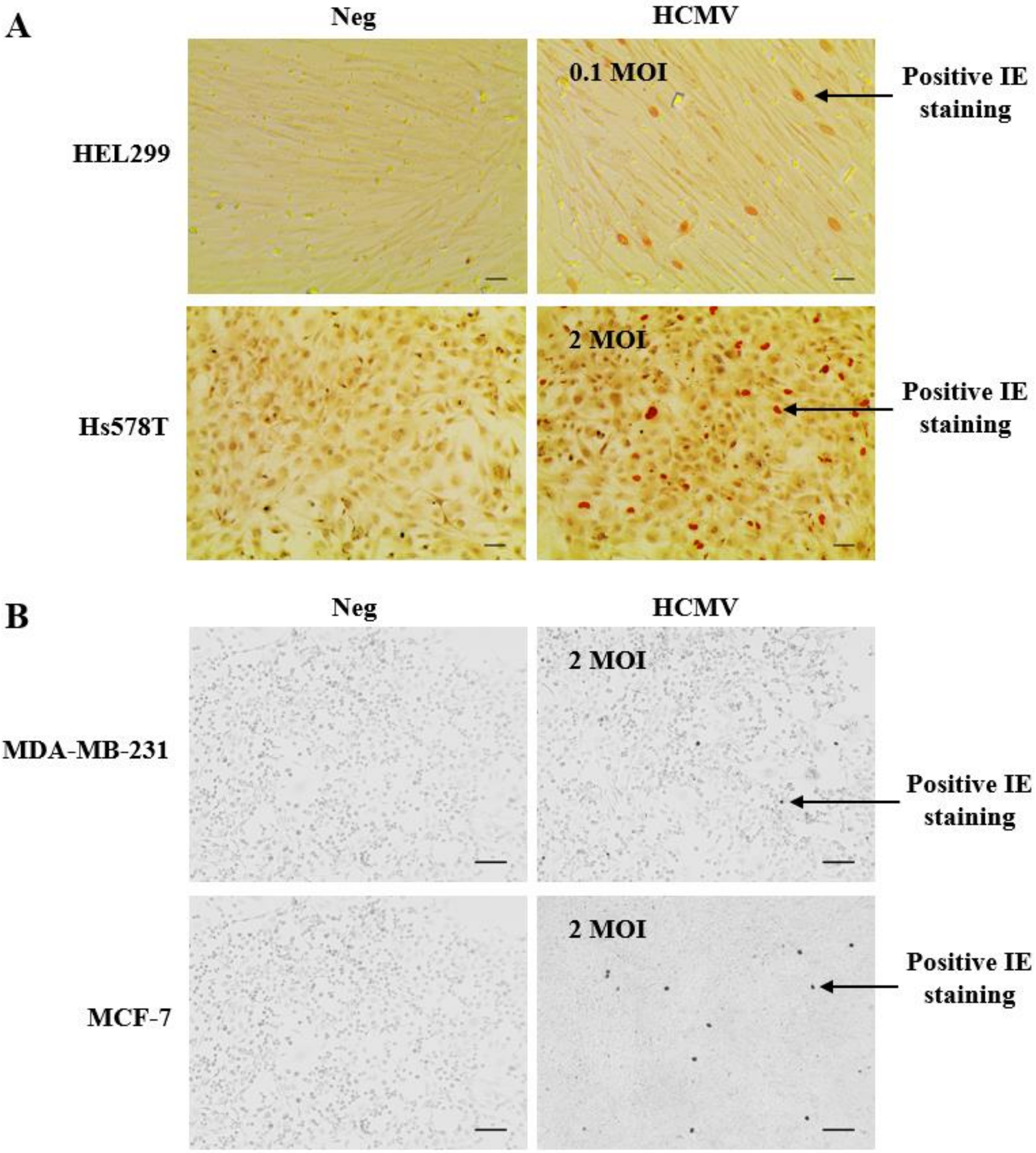

Figure S1. Immediate early (IE) antigen staining in HCMV-challenged human fibroblasts and breast cancer cells. Representative images of the cells stained for IE antigen at 36 h after initial exposure to 0.1 or 2 MOI of HCMV in HEL299 fibroblasts or breast cancer cells, respectively or to the equivalent volume of media with infectious virus filtered out as a negative control (Neg). (**A**) Images were taken at 20×. Scale = 50μm. (**B**) Images were taken at 10×. Scale = 100μm.

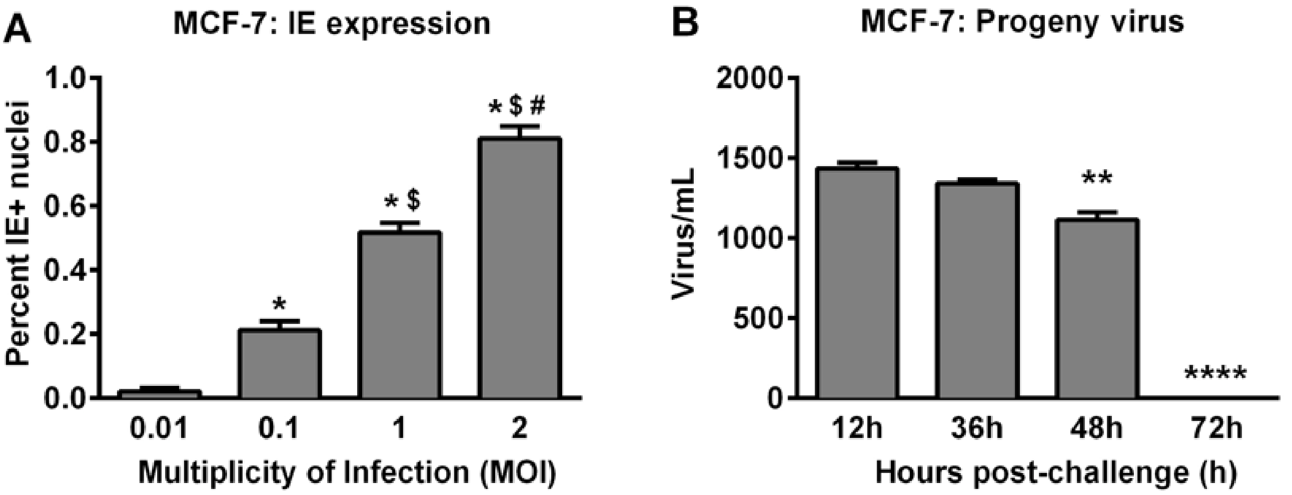

Figure S2. Limited immediate early (IE) antigen expression and production of progeny virus in MCF-7 breast cancer cells exposed to HCMV. (**A**) Cells were challenged with HCMV at MOIs of 0.01, 0.1, 1 or 2, then stained for the IE antigen at 36 h post-viral challenge to determine the percent IE-positive nuclei which were quantified using the average of 5 randomly chosen fields. Results are expressed as mean ± SEM from three independent experiments and *P*-values were calculated by one-way ANOVA with a Tukey's post-hoc test. *, $P < 0.01$ compared to 0.01 MOI; $^{\$}$, $P < 0.01$ compared to 0.1 MOI; $^{\#}$, $P < 0.05$ compared to 1 MOI. (**B**) Infectious virus was quantified from cell lysates obtained 12, 36, 48, or 72 h after exposure to 2 MOI of HCMV. Cells were washed with HEPES-buffered saline at pH 7.4 after an initial 2 h viral exposure, then further cultured in normal media with collection of lysates at each timepoint. Lysates were added to fresh uninfected HEL299 fibroblasts for 24 h and stained for HCMV IE antigen denoting infectious virus in the lysates, with results expressed as virus/mL. Results are from 3 independent experiments. *P*-values were calculated by one-way ANOVA with a Tukey's post-hoc test. **, $P < 0.01$ compared to 12 and 36 h; ****, $P < 0.0001$ compared to all other times.

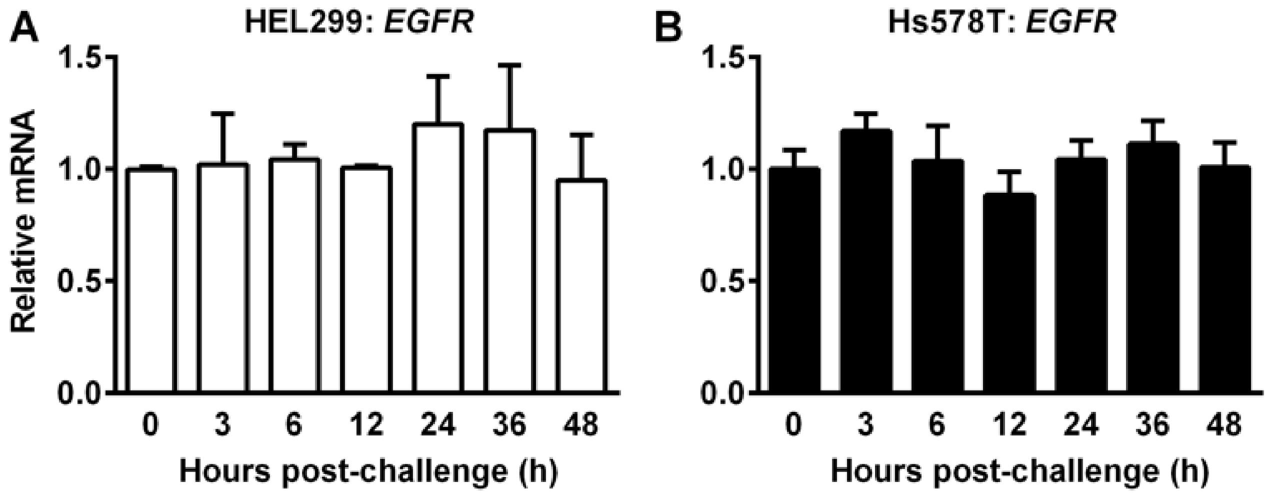

Figure S3. *EGFR* mRNA expression did not change in HEL299 or Hs578T cells exposed to HCMV. HEL299 fibroblasts (**A**) and Hs578T breast cancer cells (**B**) were exposed HCMV at 1 or 2 MOI, respectively. Lysates were collected at each timepoint and mRNA expression was measured. Results are expressed relative to cells treated with the equivalent volume of media with infectious virus filtered out as a negative control at each timepoint, and then normalized to 0 h. Results are expressed as mean ± SEM from five independent experiments and *P*-values were calculated by one-way ANOVA with no significant differences found.

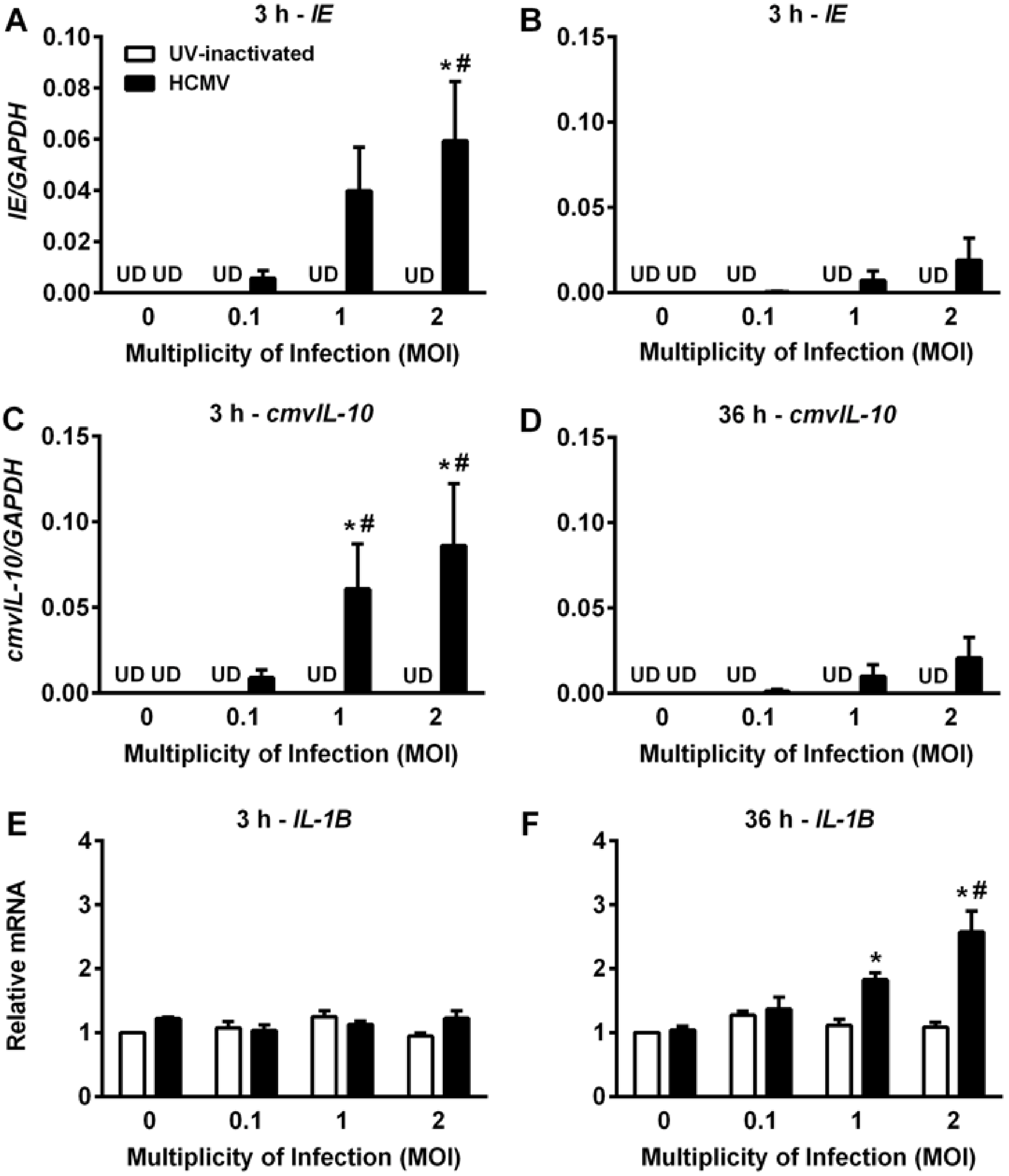

Figure S4. UV-inactivated virus did not stimulate mRNA expression of viral genes or *IL-1B* in breast cancer cells. MDA-MB-231 breast cancer cells were exposed to UV-inactivated virus or HCMV at MOIs of 0, 0.1, 1 or 2. Lysates were collected at 3 and 36 h post-challenge for mRNA expression analysis. (**A**–**D**) Expression of the viral genes was normalized against glyceraldehyde 3-phosphate dehydrogenase (*GAPDH*). (**E**) and (**F**) Expression of *IL-1B* was expressed relative to cells treated with the equivalent volume of media with infectious virus filtered out as a negative control at each timepoint, and then normalized to 0 h. Results are expressed as mean ± SEM from three independent experiments and *P*-values were calculated by two-way ANOVA and Tukey's post-hoc test. *, $P < 0.05$ compared to 0 MOI; #, $P < 0.05$ compared to UV-inactivated treatment.

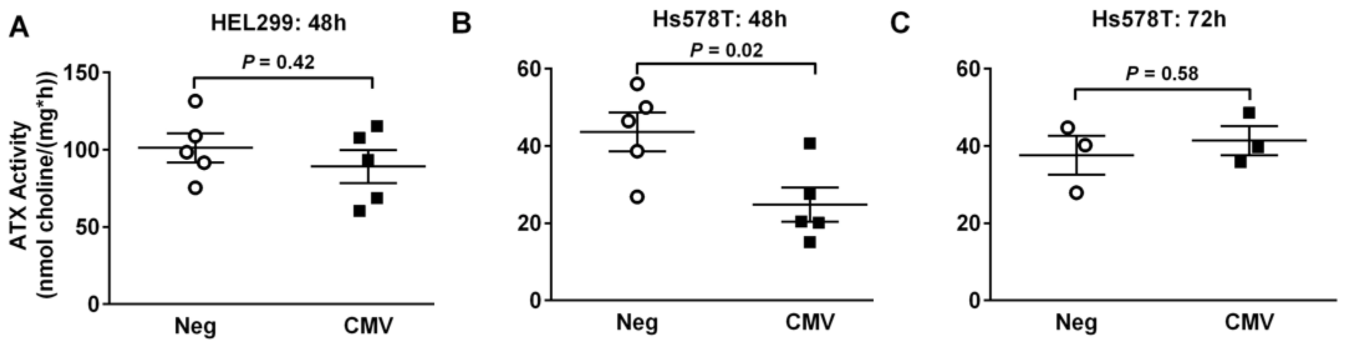

Figure S5. ATX activity was decreased in culture media from Hs578T breast cancer cells but not from HEL299 fibroblasts exposed to HCMV. Cells were exposed to HCMV at 1 or 2 MOI for HEL299 (**A**) and Hs578T (**B**,**C**)breast cancer cells, respectively or to the equivalent volume of media with infectious virus filtered out as a negative control (Neg). Culture media were collected at 48 or 72 h post-viral challenge to determine ATX activity. Results are expressed as mean ± SEM from three independent experiments and *P*-values were calculated by unpaired Student's t-test.

Table S1. Primer sequences of genes assayed by qPCR.

Gene	Primer Sequence
GAPDH	Forward: 5′-TCCTGCACCACCAACTGCTT-3′<br>Reverse:5′-TCTTACTCCTTGGAGGCCAT-3′
HCMV IE	Forward: 5′-TGAGGATAAGCGGGAGATGT-3′<br>Reverse: 5′-ACTGAGGCAAGTTCTGCAGT-3′
cmvIL-10	Forward: 5′-TCGGTGATGGTCTCTTCCTC-3′<br>Reverse:5′-CGTCGCAATAAACCGTACCT-3′
PDGFRA	Forward: 5′-TAGTGCTTGGTCGGGTCTTG-3′<br>Reverse: 5′-TTCATGACAGGTTGGGACCG-3′
EGFR	Forward: 5′-CTCTGCCTTGAGTCATCTATTC-3′<br>Reverse: 5′-CTACCTGAATTCCACACTACTC-3′
IL-1B	Forward: 5′-AGATGAAGTGCTCCTTCCAGGAC-3′<br>Reverse: 5′-TGCCTGAAGCCCTTGCTGTA-3′
IL-6	Forward: 5′-AGCCAGAGCTGTGCAGATGA-3′<br>Reverse: 5′-CTGCAGCCACTGGTTCTGTG-3′
COX-2 (PTGS2)	Forward: 5′-ATGAGTGTGGGATTTGACCA-3′<br>Reverse: 5′-ATTCCGGTGTTGAGCAGTTT-3′
ATX<br>*(ENPP2)*	Forward: 5′-CATTTATTGGTGGAACGCAGA-3′<br>Reverse: 5′-ACTTTGTCAAGCTCATTTCC-3′
LPAR2	Forward: 5′-CTGCTCATGGTGGCTGTGTA-3′<br>Reverse: 5′-TACAGCCAGGACATTGCAGG-3′
LPP1	Forward: 5′-GGTCAAAAATCAACTGCAG-3′<br>Reverse: 5′-TGGCTTGAAGATAAAGTGC-3′
LPP2	Forward: 5′-TGGCCAAGTACATGATTGG-3′<br>Reverse: 5′-AGCAGCCGTCCCCACTTCC-3′
LPP3	Forward: 5′-CCCGGCGCTCAACAACAACC-3′<br>Reverse: 5′-TCTCGATGATGAGGAAGGG-3′

Table S2. Secretion of cytokines, chemokines and growth factors for HCMV uninfected and infected HEL299 fibroblasts.

Cytokines/Chemokines	Neg Control $N = 5$ (pg/mg Protein)		CMV $N = 5$ (pg/mg Protein)		P-Value
	Mean	SEM	Mean	SEM	
**CCL1**	1.1	0.3	1.5	0.3	0.42
CCL3	4.0	0.5	55.6	28.8	0.11
CCL7	198.7	58.3	109.4	32.6	0.22
CCL8	12.9	7.0	54.5	23.3	0.13
CCL11	19.0	3.4	24.5	6.5	0.47
CX3CL1	50.7	10.9	52.6	13.2	0.92
CXCL1	381.1	289.1	22.1	6.1	0.25
CXCL5	1238.0	777.5	9.3	6.1	0.15
CXCL10	476.6	196.0	515.4	236.8	0.90
CXCL13	1.3	0.3	1.1	0.3	0.67
EGF	6.5	1.8	12.2	4.4	0.26
FGF-β	92.4	33.8	85.2	17.6	0.85
FLT-3L	2.2	0.2	3.0	0.6	0.24
G-CSF	10.4	6.6	180.1	77.3	0.06
IFNα2	13.0	2.6	19.0	3.7	0.22
IFNγ	0.9	0.1	2.4	0.7	0.06
IL-1β	5.3	1.9	5.3	1.3	0.99
IL-1RA	1.3	0.3	1.7	0.3	0.46
IL-2	0.5	0.2	0.9	0.4	0.38
IL-8	2626.0	1849.0	8356.0	2128.0	0.08
IL-9	4.0	0.7	6.2	2.0	0.35
IL-10	2.0	0.3	2.0	0.4	0.96
IL-12p40	19.3	2.0	28.6	6.9	0.23
IL-13	22.8	4.8	11.4	4.3	0.11
IL-15	26.1	3.3	30.5	6.0	0.54
IL-17A	0.9	0.5	2.3	1.2	0.28
IL-17F	0.6	0.3	3.8	2.0	0.16
IL-18	0.4	0.0	0.5	0.1	0.41
IL-21	2.0	0.5	2.1	0.9	0.95
IL-22	39.2	11.2	42.7	11.0	0.83
IL-23	45.5	21.6	67.8	28.7	0.55
IL-27	32.7	14.0	53.8	28.0	0.52
IL-33	5.2	1.9	4.6	1.9	0.83
LIF	174.0	100.8	56.8	15.5	0.28
M-CSF	424.0	17.9	446.2	126.5	0.87
PDGF-AA	9.1	1.3	14.3	2.5	0.10
PDGF-AB/BB	33.0	8.4	30.1	13.2	0.86
sCD40L	17.3	4.2	23.1	5.7	0.43
TGFα	1.7	0.3	3.0	1.0	0.22
TNFβ	18.5	3.9	26.0	7.6	0.40
TRAIL	1.9	0.5	2.5	0.6	0.44

Table S3. Secretion of cytokines, chemokines and growth factors for HCMV uninfected and infected Hs578T breast cancer cells.

Cytokines/Chemokines	Neg Control *N* = 5 (pg/mg Protein)		CMV *N* = 5 (pg/mg Protein)		*P*-Value
	Mean	SEM	Mean	SEM	
**CCL1**	0.8	0.2	0.5	0.0	0.24
CCL2	116980.0	54509.0	26232.0	15880.0	0.15
CCL3	1.6	1.0	1.4	0.8	0.85
CCL8	20.4	6.4	11.0	4.8	0.28
CCL11	99.0	47.5	149.8	123.8	0.71
CCL26	69.4	34.5	0.0	0.0	0.08
CXCL5	17.3	5.9	7.2	3.8	0.19
CXCL9	3.5	0.8	2.2	0.4	0.20
CXCL13	1.4	0.4	1.1	0.3	0.46
FGF-β	2136.0	1446.0	29.0	4.7	0.22
GM-CSF	18.4	5.7	17.5	6.0	0.92
IL-1α	18.0	8.8	4.1	0.8	0.16
IL-1RA	9.2	4.8	1.0	0.2	0.13
IL-6	11561.0	3149.0	10197.0	2366.0	0.74
IL-9	4.9	1.2	2.4	0.5	0.09
IL-10	1.5	0.4	0.9	0.2	0.23
IL-15	25.9	10.0	4.9	1.9	0.07
IL-16	5.9	2.9	0.6	0.6	0.11
IL-17F	3.9	1.5	1.4	0.3	0.14
IL-20	373.8	156.7	334.0	90.4	0.83
IL-23	73.4	29.5	63.8	32.6	0.83
IL-28A	4.0	2.1	1.6	0.8	0.32
IL-33	5.5	1.6	5.3	1.5	0.93
LIF	12.7	7.2	1.7	0.7	0.16
TPO	3.8	2.8	5.6	4.1	0.72
TRAIL	1.5	0.4	1.2	0.3	0.58

Table S4. Secretion of cytokines, chemokines and growth factors for HCMV uninfected and infected MDA-MB-231 breast cancer cells.

**Cytokines/Chemokines**	**Neg Control *N* = 3 (pg/mg Protein)**		**CMV *N* = 3 (pg/mg Protein)**		***P*-Value**
	**Mean**	**SEM**	**Mean**	**SEM**	
**CCL1**	0.9	0.0	0.8	0.1	0.68
CCL2	14.9	9.2	21.2	8.5	0.64
CCL5	25.7	14.5	30.1	6.6	0.80
CCL22	4.7	1.9	5.4	1.5	0.78
CCL24	2.1	1.4	1.0	0.9	0.56
CXCL5	35.1	8.3	8.2	8.2	0.08
CXCL9	4.4	0.7	4.2	0.5	0.82
CXCL10	10.8	8.0	10.8	2.1	0.99
CXCL13	1.7	0.2	2.0	0.2	0.32
FLT-3L	0.7	0.2	0.8	0.1	0.77
IFNγ	0.6	0.1	0.6	0.0	0.76
IL-1β	0.5	0.4	1.4	0.8	0.40
IL-1RA	1.0	0.1	2.9	2.3	0.45
IL-4	0.4	0.2	0.5	0.1	0.78
IL-6	58.1	22.0	61.7	31.2	0.93
IL-8	480.8	456.9	498.2	453.4	0.98
IL-9	2.0	1.0	3.5	0.3	0.23
IL-10	1.5	0.1	1.5	0.0	0.84
IL-12p40	1.9	0.7	3.1	0.2	0.18
IL-13	4.2	2.4	1.3	1.3	0.35
IL-15	0.7	0.3	0.9	0.4	0.69
**IL-17F**	**0.2**	**0.2**	**1.3**	**0.2**	**0.02**
IL-20	649.1	123.2	467.6	54.0	0.25
IL-21	5.2	1.0	4.0	0.8	0.40
IL-23	123.7	38.3	114.7	27.4	0.86
IL-28A	5.0	0.6	3.9	1.2	0.42
IL-33	8.5	0.6	7.9	1.0	0.64
M-CSF	22.6	15.4	27.9	8.9	0.78
PDGF-AA	93.8	50.1	143.9	32.0	0.45
PDGF-AB/BB	93.6	67.7	180.6	45.4	0.35
SCF	9.3	0.5	9.0	2.6	0.92
TGFα	5.1	2.0	8.6	0.6	0.16
TNFα	2.8	2.1	4.2	1.1	0.60
TNFβ	1.8	0.8	1.9	0.8	0.96
TRAIL	3.2	0.3	3.0	0.1	0.59
VEGF-A	49.2	26.7	53.2	17.5	0.91
